# Cardiac arrest in the perioperative period: a consensus guideline for identification, treatment, and prevention from the European Society of Anaesthesiology and Intensive Care and the European Society for Trauma and Emergency Surgery

**DOI:** 10.1007/s00068-023-02271-3

**Published:** 2023-07-10

**Authors:** Jochen Hinkelbein, Janusz Andres, Bernd W. Böttiger, Luca Brazzi, Edoardo De Robertis, Sharon Einav, Carl Gwinnutt, Bahar Kuvaki, Pawel Krawczyk, Matthew D. McEvoy, Pieter Mertens, Vivek K. Moitra, Jose Navarro-Martinez, Mark E. Nunnally, Michael O´Connor, Marcus Rall, Kurt Ruetzler, Jan Schmitz, Karl Thies, Jonathan Tilsed, Mauro Zago, Arash Afshari

**Affiliations:** 1 Department of Anaesthesiology, Intensive Care Medicine and Emergency Medicine, Johannes Wesling Klinikum Minden, Ruhr-University Bochum, Minden, Germany; 2 Department of Anaesthesiology and Intensive Care Medicine, Medical Faculty, University Hospital of Cologne, Kerpener Str. 62, 50937 Cologne, Germany; 3 Department of Anaesthesiology and Intensive Therapy, Jagiellonian University Medical College, Krakow, Poland; 4 The Department of Surgical Sciences, University of Turin, Turin, Italy; 5 The Division of Anaesthesia, Analgesia and Intensive Care, Department of Medicine and Surgery, University of Perugia, Perugia, Italy; 6 The Intensive Care Unit, Shaare Zedek Medical Center, Hebrew University Faculty of Medicine, Jerusalem, Israel; 7 The Department of Anaesthesia, Salford Royal NHS Foundation Trust, Salford, UK; 8 The Department of Anesthesiology and Reanimation, Dokuz Eylül University, İzmir, Turkey; 9 The Department of Anesthesiology and Intensive Care Medicine, Jagiellonian University Medical College, Krakow, Poland; 10 The Department of Anaesthesiology, Antwerp University Hospital, Drie Eikenstraat 655, 2650 Edegem, Belgium; 11 The Department of Anesthesiology, Vanderbilt University Medical Center, Nashville, TN USA; 12 Division of Critical Care Anesthesiology, The Department of Anesthesiology, Columbia University, Columbia, NY USA; 13 The Anesthesiology Department, Dr. Balmis General University Hospital, Alicante Institute for Health and Biomedical Research (ISAB), Biomedical Research (ISABIAL), Alicante, Spain; 14 The Department of Anesthesiology, Perioperative Care, and Pain Medicine, NYU Grossman School of Medicine, New York, NY USA; 15 The Department of Anesthesiology & Critical Care, University of Chicago, Chicago, IL USA; 16 The Institute for Patient Safety and Simulation Team Training InPASS, Reutlingen, Germany; 17 The Departments of General Anesthesiology and Outcomes Research, Anesthesiology Institute, Cleveland Clinic, Cleveland, OH USA; 18 The Department of Anaesthesiology and Critical Care, EvKB, OWL University Medical Center, Bielefeld University, Campus Bielefeld-Bethel, Bethel, Germany; 19 The Department of Surgery, Hull University Teaching Hospitals, Hull, UK; 20 General & Emergency Surgery Division, The Department of Surgery, A. Manzoni Hospital, Milan, Italy; 21 The Department of Paediatric and Obstetric Anaesthesia, Copenhagen University Hospital, Rigshospitalet, Denmark

**Keywords:** Cardiac arrest, Operating room, Resuscitation, Cardiopulmonary resuscitation (CPR)

## Abstract

**Introduction:**

Cardiac arrest in the operating room is a rare but potentially life-threatening event with mortality rates of more than 50%. Contributing factors are often known, and the event is recognised rapidly as patients are usually under full monitoring. This guideline covers the perioperative period and is complementary to the European Resuscitation Council guidelines.

**Material And Methods:**

The European Society of Anaesthesiology and Intensive Care and the European Society for Trauma and Emergency Surgery jointly nominated a panel of experts to develop guidelines for the recognition, treatment, and prevention of cardiac arrest in the perioperative period. A literature search was conducted in MEDLINE, EMBASE, CINAHL and the Cochrane Central Register of Controlled Trials. All searches were restricted to publications from 1980 to 2019 inclusive and to the English, French, Italian and Spanish languages. The authors also contributed individual, independent literature searches.

**Results:**

This guideline contains background information and recommendation for the treatment of cardiac arrest in the operating room environment, and addresses controversial topics such as open chest cardiac massage, resuscitative endovascular balloon occlusion and resuscitative thoracotomy, pericardiocentesis, needle decompression, and thoracostomy.

**Conclusions:**

Successful prevention and management of cardiac arrest during anaesthesia and surgery requires anticipation, early recognition, and a clear treatment plan. The ready availability of expert staff and equipment must also be taken into consideration. Success not only depends on medical knowledge, technical skills and a well-organised team using crew resource management, but also on an institutional safety culture embedded in everyday practice through continuous education, training, and multidisciplinary co-operation.

**Supplementary Information:**

The online version contains supplementary material available at 10.1007/s00068-023-02271-3.

## Introduction

Cardiac arrest in the perioperative period is a rare but potentially life-limiting event with mortality rates of more than 50% [1, 2]. Data collected from 250 United States hospitals (1.3 million surgical cases) found that one out of 203 surgical patients undergo cardiopulmonary resuscitation (CPR). This occurs more often during cardiac surgery than general surgery (1 in 33 v 1 in 258) and was associated with a mortality of more than 50% within the first 30 postoperative days [2, 3].

Contributing factors are often known in advance and the event is generally recognised rapidly, as patients are usually fully monitored. The cause of cardiac arrest in the operating room is often different from other environments because it is related to the patients’ conditions and may result from both the anaesthetic technique and the complexity of the surgical procedure [2]. Compared to cardiac arrest in general, that occurring in the operating room environment is characterised by reversible causes and the presence of trained staff and enhanced resources. As with the out-of-hospital environment [4], where outcomes can be improved with training in resuscitation protocols, cardiac arrest in the perioperative period may be amenable to enhanced recommendations and training. However, cardiac arrest in this period is still considered ‘the poor relation’ of CPR [5] because protocols are designed for out-of-hospital arrest (Table 1).

**Table 1:** Summary of recommendations

	Topic	Recommendation	Evidence
Identification of cardiac arrest	Monitoring	Studies evaluating standard and haemodynamic monitoring are animal based. These are not comparable with human studies	Recommendations based on expert opinion only
		Use of end-tidal CO_2_ monitoring in intubated patients during CPR may help to predict the likelihood of return of spontaneous circulation and survival as well as guiding CPR, despite the lack of absolute cut-off values	Weak recommendation moderate quality evidence (2B)
		Invasive blood pressure monitoring during closed chest compression could potentially improve quality and optimise the timing of adrenaline administration	Weak recommendation, low quality evidence (2C)
		Besides standard (and invasive) monitoring, if the expertise is present, the equipment available, and the patient’s condition allows it, transoesophageal echocardiography is suggested during a peri-procedural cardiac arrest to identify the aetiology of the arrest and guide further management	Weak recommendation, low-quality evidence (2C)
Management of cardiac arrest		We recommend closed chest cardiac compression for patients with cardiac arrest	Strong recommendation, low quality evidence (1C)
		Open chest cardiac massage should be considered if return of spontaneous circulation has not been achieved with closed chest cardiac compression and VA-ECMO is not available	Weak recommendation, low quality evidence (2C)
Specific management of complications during surgery	Gas embolism during laparoscopy	We recommend closed chest cardiac compression for patients with a gas embolism who develop a cardiac arrest	Strong recommendation, low quality evidence (1C)
		Open chest cardiac massage should be considered if return of spontaneous circulation has not been achieved with closed chest cardiac compression and VA-ECMO is not available	Weak recommendation, low quality evidence (2C)
	Pulmonary Embolism	VA-ECMO should be considered for restoring circulation and oxygenation as a bridge to definitive treatment. Also consider thrombolysis if extra-corporeal membrane oxygenation is not available	Strong recommendation, low quality evidence (1C)
	Pulseless rhythms	Cardiac arrest presenting with ventricular fibrillation in the perioperative setting should be treated with immediate defibrillation	Strong recommendation, moderate quality evidence (1B)
		Cardiac arrest presenting with pulseless ventricular tachycardia in the perioperative setting should be treated with immediate defibrillation	Strong recommendation, moderate quality evidence (1B)
		For asystole with p-waves, emergency temporary pacing should be performed. Reversible causes should be addressed without delay	Weak recommendation, moderate‐quality evidence (2B)
	Haemorrhage	We suggest simultaneous haemorrhage control, massive transfusion, and closed chest compressions	Strong recommendation, low quality evidence (1C)
		We suggest simultaneous volume replacement and closed chest compressions	Weak recommendation, low quality evidence (2C)
		If there is no return of spontaneous circulation despite adequate volume therapy, open chest cardiac massage may be considered	Weak recommendation, low‐quality evidence (2C)
	Resuscitative endovascular balloon occlusion of the aorta and resuscitative thoracotomy	In patients with exsanguinating infra-diaphragmatic haemorrhage uncontrollable by other means we suggest immediate use of resuscitative endovascular balloon occlusion of the aorta	Weak recommendation, low quality evidence (2C)
		We suggest the use of either resuscitative thoracotomy with cross-clamping of the descending aorta or resuscitative endovascular balloon occlusion of the aorta	Weak recommendation, low quality evidence (2C)
	Tension pneumothorax	We recommend immediate decompression of suspected tension pneumothorax	Strong recommendation, low quality evidence (1C)
		We recommend needle decompression immediately if tension pneumothorax is the proven or suspected cause of the cardiac arrest	Strong recommendation, low quality evidence (1C)
		We recommend finger thoracotomy or a chest tube insertion after any needle decompression attempt	Strong recommendation, low quality evidence (1C)
	Cardiac tamponade	In suspected cardiac tamponade, point of care ultrasound should be used to confirm the diagnosis	Strong recommendation, low-quality evidence (1C)
Procedures	CCC or OCCM	We recommend closed chest compressions for patients with cardiac arrest	Strong recommendation, low quality evidence (1C)
		Open chest cardiac massage may be considered if return of spontaneous circulation has not been achieved with closed chest compressions and if VA-ECMO not available	Weak recommendation, low quality evidence (2C)
	Pericardiocentesis	In the case of cardiac tamponade, we recommend immediate decompression of the pericardium	Strong recommendation, low-quality evidence (1C)
		Immediate decompression can be achieved by either ultrasound guided pericardiocentesis or, in the case of a haemopericardium, by resuscitative thoracotomy	Strong recommendation, low-quality evidence (1C)
Preparational aspects of cardiac arrest	Cardiac arrest team training	When training for peri-operative cardiac arrest, we suggest a co-ordinated protocol to improve the quality of mechanical cardiopulmonary resuscitation	Weak recommendation, low‐quality evidence (2C)
		We suggest simulation training since experience and training of healthcare providers increases the likelihood of return of spontaneous circulation	Weak recommendation, low‐quality evidence (2C)

*CPR* cardio pulmonary resuscitation, *VA-ECMO* Veno-arterial extracorporeal membrane oxygenation, *CCC* closed chest compression, *OCCM* open chest cardiac massage

This evidence-based guideline aims to complement the guidelines of the European Resuscitation Council (ERC) and provide specific recommendations for the identification, treatment and prevention of cardiac arrest in the perioperative setting [6, 7]. As well as its focus on the time in the operating room, it includes the immediate pre-operative phase, anaesthesia induction, and the post-operative period in the recovery room (Table 2).

**Table 2:** Clinical best practice statements

Management of specific causes	Hypovolaemic cardiac arrest	Point of care ultrasound for assessment of volume and myocardial contractility has the potential to target resuscitative efforts in a cardiac arrest situation
Prevention	Training of healthcare providers	We recommend simulation training since experience and training of healthcare providers increases the likelihood of return of spontaneous circulation

## Material and methods

The European Society of Anaesthesiology and Intensive Care (ESAIC) and the European Society of Trauma and Emergency Surgery (ESTES) each nominated a panel of experts to develop guidelines for the identification, treatment, and prevention of perioperative cardiac arrest.

Following several rounds of discussions and voting during meetings of these two expert panels commencing in 2017, 26 different questions were identified that required answers by the guideline. These clinical questions were developed into 32 population, intervention, comparison, outcomes (PICO) questions that laid the groundwork for the design of the search strategy.

### Objectives

The objective was to evaluate the available literature on the prevention, identification, and treatment of peri-operative cardiac arrest. This objective was approved in 2018 by the boards of ESAIC and ESTES. Delegates from the European Board of Anaesthesiology and the American Society of Anaesthesiologists also took part in the working group.

### Definitions

Data analysed described investigation in the perioperative period and were limited to adult patients. In cases of little or no evidence in the perioperative period, data from other settings (e.g., pre-hospital; in-hospital) were also used.

### Criteria for considering studies for data analysis

Types of studies: data analysis included all randomised, parallel, and observational (including cross-over) studies performed in adult humans comparing any of the above criteria for perioperative cardiac arrest. Data from observational studies were included due to the small number of randomised controlled trials. Retrospective studies, reviews, case series and case reports were excluded unless data were lacking altogether, in which case retrospective data and expert knowledge were used to derive an expert opinion. Similarly, when perioperative or periprocedural data were lacking, information was extrapolated from data in other settings.

Types of patients: the qualitative and quantitative analysis of the literature was limited to adult patients having cardiac arrest in the perioperative period. Studies relating solely to paediatric patients were excluded due to the differences in physiology and clinical approach to management of perioperative cardiac arrest in this age group. Studies including paediatric and adult populations were reviewed if the majority were adult patients.

### Types of interventions

We included the following topics (as agreed by the authors) as the clinical interventions:

Clinical interventions.

- Management of coagulopathy
- Bleeding triggers
- Resuscitative thoracotomy
- Cardiac compressions
- Trendelenburg positioning
- Extracorporeal cardiopulmonary resuscitation
- Thrombolysis
- Embolectomy
- Chest compression
- Open cardiac massage
- Resuscitative thoracotomy
- Resuscitative endovascular balloon occlusion of the aorta
- Decompression
- Needle pericardiocentesis
- Transthoracic echocardiography
- Transoesophageal echocardiography
- End tidal carbon dioxide
- Immediate cardiac catheterisation
- Withdrawing therapy in the operating room
- Effect of prior skilled training
- Simulation
- Structured communication by team-leader and within the team

Types of comparators.

- Standard surgical/anaesthetic care.
- Not performing specific therapy (e.g., extracorporeal membrane oxygenation, thrombolysis).
- Chest compression only.
- Delayed treatment in the catheter laboratory.

Types of outcomes: the focus was preferentially on clinical outcomes, e.g., return of spontaneous circulation (ROSC), length of survival post-cardiac arrest, hospital discharge, mortality, long-term neurological outcome.

### Search methods for identification of studies

The panel was divided into subgroups and each was allocated one of the 32 questions. Each subgroup formulated the relevant questions and suggested keywords for their literature search. The list of questions and the accompanying keywords were sent to the entire panel for discussion, amendment, and approval. The final list of keywords framed the literature search.

### Electronic searches

The literature search strategy was developed by a Cochrane Anaesthesia and Intensive Care trial search specialist (Copenhagen, Denmark) in close collaboration with the panel of experts, the ESAIC group methodologist and Cochrane editor (AA). The literature search was conducted using MEDLINE (OvidSP), EMBASE (OvidSP), CINAHL and the Cochrane Central Register of Controlled Trials (CENTRAL). All searches were restricted to English, French, Italian or Spanish languages and from the beginning of 1980 to the end of 2019. A similar search strategy was used for all the databases and repeated twice for 2019 data. The panel members were also encouraged to add any missing paper of interest of which they were aware and to conduct a “snow-balling” search themselves.

After removal of all duplicates, the authors screened the abstracts and titles. All relevant papers were retrieved for full-text assessment and data extraction. The decision to carry out any meta-analysis was made after close discussions with the methodologist based on the quality of the available data, reliability of the search (sensitivity) and predefined inclusion and exclusion criteria. We found no data suitable for meta-analysis for this guideline.

### Additional resources

For trials not yet completed, a search was conducted in clinical trials registries (clinicaltrials.gov; controlled-trials.com; anzctr.org.au; and who.int/ictrp). Eligible trials were also screened for additional, and previously unidentified studies. The following were not sought; published abstracts from conference proceedings of any society or new studies of potential interest. Trial authors were not contacted to determine whether any additional data was pending. The objective was to search for online studies that were finalised with potential for inclusion. All authors of these guidelines were advised to provide any missing relevant articles that were not included in the first round in order to increase the precision of the search and revise the search strategy accordingly. Additional references of importance published after the literature search were also included.

### Data collection and analysis

#### Selection of studies

All papers meeting inclusion criteria were included. At least two authors within each of the 32 PICO subgroups examined independently the titles and abstracts of the articles identified during the search and screened them for suitability. Disagreements were resolved by third party adjudication. If relevant, the full text was assessed.

#### Data extraction and management

Each pair of review authors extracted data from relevant studies onto a predesigned Excel data extraction table consisting of: study design, population characteristics, interventions, and outcome measures. Review authors reached consensus regarding extracted data through discussion.

#### Assessment of risk of bias in included studies

Review authors assessed the risk of bias of each of the studies selected for their PICO question. Risk of bias assessment was conducted in accordance with the Cochrane Handbook for Systematic Reviews of Interventions (Version 6.1) [8] and was assessed for the following domains:

- Random sequence generation (selection bias)
- Allocation concealment (selection bias)
- Blinding of outcome assessors (performance and detection bias)
- Incomplete outcome data, intention-to-treat (attrition bias)
- Reporting bias

Trials were assessed as having a low risk of bias if all domains were considered adequate. A high risk of bias was considered if one or more of these domains was inadequate or unclear.

### Assessment of quality of the evidence

In accordance with ESAIC policy, grading of recommendations, assessment, development and evaluation (GRADE) methodology (Appendix 1) was used for assessing the level of evidence of the included studies and for formulating the recommendations.

Decisions to downgrade the level of evidence for a recommendation were based on the quality and type of literature, observed inconsistencies, indirectness of the evidence, overall imprecision and the probability of publication bias by GRADE. Decisions to upgrade the level of evidence for recommendations were based on study quality and magnitude of effect, dose–response gradient, and plausible confounding.

### Development of recommendations

Each subgroup developed recommendations relevant to their PICO questions. These were then discussed and re-discussed as required with the expert panel based on the data (when available), the risk of bias and the quality of the evidence. Each draft and its revisions were reviewed by the panel and the final version was approved by all members. After agreement with the final terminology, the recommendations were merged by the first author into a shared document and the final version was revised and approved by all panel members.

## Results

### Identification of cardiac arrest

#### Monitoring

- Studies evaluating standard and haemodynamic monitoring are animal based. These are not comparable with human studies.

Recommendations based on expert opinion.

- The use of end-tidal CO2 monitoring in intubated patients during CPR may help to predict the likelihood of return of spontaneous circulation and survival as well as guiding CPR despite the lack of absolute cut-off values

Weak recommendation, moderate quality evidence (2B).

- Invasive blood pressure monitoring during closed chest compression could potentially improve quality and optimise the timing of adrenaline administration.

Weak recommendation, low quality evidence (2C).

- Besides standard (and invasive) monitoring, if the expertise is present, the equipment available, and the patient’s condition allows it, transoesophageal echocardiography is suggested during a peri-procedural cardiac arrest to identify the aetiology of the arrest and guide further management.

Weak recommendation, low-quality evidence (2C)

The impact of standard and invasive haemodynamic monitoring on the outcome of adult patients who had CPR in the operating room were evaluated. Several animal studies have found that haemodynamic guided resuscitation improves survival [9–13]. There are no similar human studies evaluating the impact of monitoring on survival.

Intraoperative studies of the predictive value of ETCO_2_ (end tidal carbon dioxide) in intubated patients during circulatory collapse have found that low levels (< 20 mm Hg) are more common with severe anaphylaxis and are associated with non-survival during emergency trauma surgery [14, 15]. Data from a systematic review suggests that low ETCO_2_ values during CPR may reflect a reduced incidence of ROSC and survival but also highlighted the lack of any absolute cut-off values [16]. Measurement of ETCO_2_ in pre-hospital cardiac arrest correlates well, with the chance of ROSC during CPR. [7]

A simulation study found that residents who were provided with invasive blood pressure monitoring were quicker to palpate pulses, initiate chest compressions, and administer adrenaline than residents who were provided with only non-invasive blood pressure monitoring [17]. A different study demonstrated that the presence of continuous arterial blood pressure monitoring resulted in improved quality of compressions during simulated cardiac arrest. [18]

Transoesophageal echocardiography has been used to identify the aetiology of intraoperative cardiac arrest and guide management in several observational case series. So far, no study has assessed the impact of TOE on survival [19–22]. There are similar data from observational studies performed mostly in emergency settings regarding the value of echocardiography for diagnosis of the aetiology of cardiac arrest but not for improving outcomes [23]. One systematic review suggested that the absence of spontaneous cardiac motion seen with TOE in patients with a a low probability for ROSC may predict a low likelihood of survival as well as guide CPR decisions, but the clinical settings of the included studies were outside of the operating room. [24]

### Management of cardiac arrest

#### Closed chest compression and open chest cardiac massage

- We recommend closed chest cardiac compression for patients with cardiac arrest.
- Strong recommendation, low quality evidence (1C)
- Open chest cardiac massage should be considered if return of spontaneous circulation has not been achieved with closed chest cardiac compression and Veno-arterial extracorporeal membrane oxygenation is not available.
- Weak recommendation, low quality evidence (2C)

Evidence is based on animal data and expert opinion supporting the re-introduction of Open chest cardiac massage (OCCM) for treatment of cardiac arrest in the operating room (OR) and the perioperative environment if ROSC cannot be achieved within minutes by advanced life support (ALS), particularly after addressing the reversible causes.

OCCM used to be the standard of care [75, 76] until closed chest compression was introduced into clinical practice in 1961 [77]. When OCCM is employed within the first minutes of cardiac arrest, hospital discharge rates of up to 50% were achieved [78]. The efficiency of Closed chest compressions (CCC) was questioned [79] and animal experimental research into the topic revealed that under OCCM cardiac output, cardiac index, coronary perfusion pressure (corPP), carotid artery flow and cerebral perfusion were significantly better than under closed chest compressions [80–89]. This correlates with significantly higher ROSC rates, long term survival, less cerebral tissue damage and better neurological function in survivors [47, 80, 81, 89–92]. Case series and observational studies in humans confirmed the experimental findings; cardiac index [93], corPP [94], ROSC rate and hospital discharge rate [95] were significantly better in patients treated with OCCM compared to patients receiving closed chest compressions. Due to the higher cardiac output achieved under OCCM metabolic deterioration does not develop as fast as under closed chest compressions [96], which justifies longer resuscitation attempts to address reversible causes. The typical access to the heart is via a left antero-lateral thoracotomy, which can be accomplished within a minute but requires a trained team. If cardiac arrest occurs during laparotomy, subdiaphragmatic or transdiaphragmatic massage are possible [97].

In traumatic cardiac arrest due to blunt trauma there is conflicting evidence regarding the survival benefit of OCCM over chest compressions [44, 98, 99]. For the treatment of traumatic cardiac arrest (TCA), we refer to the corresponding algorithm of the ERC [6] and the European Trauma Course (http://www.europeantraumacourse.com).

Even if appropriate resuscitative manoeuvres for critical blunt trauma patients remain somewhat unclear, retrospective cohort studies suggest that the great majority of emergency resuscitative thoracotomies in this patient population were inappropriate, incurred substantial expense with an increased risk of exposure of health-care workers to possible blood-borne infections and no survival benefits. It has been observed that the effectiveness of emergency resuscitative thoracotomy for trauma patients depends on the time from cardiac arrest to the procedure.

### Management of complications during surgery

#### Gas embolism

- We recommend closed chest cardiac compression for patients with a gas embolism who develop a cardiac arrest.
- Strong recommendation, low certainty evidence (1C)
- Open chest cardiac massage should be considered if return of spontaneous circulation has not been achieved with closed chest cardiac compression and VA-ECMO is not available.
- Weak recommendation, low quality evidence (2C)

Evidence supporting interventions for gas embolism is limited (case reports and indirect animal data). Head-down, and left side-down positioning would seem reasonable to prevent air passing into the right ventricular outflow tract. Head-down [25, 26] and left-side down [27] positions are supported by canine studies, and described in human case reports [28]. Benefits include improved haemodynamics [25, 27], improved time to resuscitation [26] and survival [26, 28]. Other canine studies comparing ten-degree head-down positioning combined with left-side down positioning demonstrated no beneficial effect on haemodynamics [29, 30]. However, these studies used a slow air injection rate (2.5 ml kg^−1^ injected at 5 ml sec^−1^) compared to larger boluses in the positive studies.

Some animal studies [25, 27, 29] do support the physiological rationale for cardiovascular collapse that responds to position change. There is a lack of evidence supporting head-down or possible left-side down positioning despite a perceived physiological benefit.

In animal models, cardiovascular collapse occurs when the right ventricle is no longer capable of overcoming the increased pulmonary vascular resistance that results from gas embolism. In circulatory arrest, it can be assumed that closed chest compressions would be beneficial to support the circulation.

Case reports highlight sources of gas embolism from hysteroscopy [31], laparoscopy [32] and lung insufflation for segmentectomy [33]. Although not related to gas insufflation, air embolism during neurosurgery is more common if the venous sinuses are opened with the head elevated above the level of the heart and it is maximal in the sitting position [117, 118]. Air embolism has also been reported during supine infratentorial intracranial surgery [119, 120] and spinal surgery [121].

Transthoracic needle lung biopsy is also an additional source for intravascular gas [34]. Circulation was supported by closed chest compressions [32], OCCM [33] and cardiopulmonary bypass [31], all with survival and at least partial recovery.

In the setting of suspected gas embolism, several interventions may be appropriate. Ceasing insufflation is crucial. Finding and stopping sources of air entrainment (e.g., open venous sinuses, tracts from lung biopsy, exteriorised uterine vessels) and flooding the surgical field with saline or lactated Ringer’s solution can reduce gas entrainment.

Supportive measures during cardiac arrest include CPR, which may disrupt gas bubbles and improve the circulation, and pressors/inotropes. Even with low quality evidence, we feel that the relative benefit of closed chest compressions is so disproportionate to not giving them that the recommendation should be a strong one. Given that right heart failure may precipitate shock and cardiac arrest, drugs that maintain arterial pressure may help relieve haemodynamic compromise. This benefit may be due to improved blood flow to the right ventricle, which occurs in systole and diastole and is therefore sensitive to systemic hypotension. Patients recovering from suspected gas embolism should receive a high-inspired oxygen concentration to facilitate absorption of intravascular gas.

### Pulmonary embolism

- VA-ECMO should be considered for restoring circulation and oxygenation as a bridge to definitive treatment. Also consider thrombolysis if extra-corporeal membrane oxygenation is not available.
- Strong recommendation, low quality evidence (1C)

VA-extracorporeal life support in peri-operative cardia arrest caused by massive pulmonary embolism can be lifesaving, restoring circulation and oxygenation whilst definitive treatment is being organised. The evidence is based on case reports [35, 36] and a small case series [37] reporting a favourable outcome of massive pulmonary embolism with the use of early VA- ECMO support in the OR or in the immediate perioperative period.

Thrombolysis is the first line treatment in massive pulmonary embolism. In the perioperative setting however, thrombolysis has the potential to cause increased and possibly fatal haemorrhage and should therefore be used only after carefully balancing the risks against the intended benefits [38]. VA-ECMO is a recognised treatment option [39]. The introduction of heparin-bonded circuits [38] has eliminated the need for intravenous anticoagulation, minimising the risk of bleeding, and makes VA-EVMO a valid option in cardiac arrest or peri-arrest situation to allow for definitive treatment.

Besides VA-ECMO, intraoperative lysis should be considered and balanced against the risk of massive haemorrhage. Thrombolysis should be considered in the perioperative period as previous data from the out-of-hospital setting reported benefit [40, 41].

### Pulseless rhythms

- Cardiac arrest presenting with ventricular fibrillation in the perioperative setting should be treated with immediate defibrillation.
- Strong recommendation, moderate quality evidence (1B)
- Cardiac arrest presenting with pulseless ventricular tachycardia in the perioperative setting should be treated with immediate defibrillation.
- Strong recommendation, moderate quality evidence (1B)
- For asystole with p-waves, emergency temporary pacing should be performed. Reversible causes should be addressed without delay.
- Weak recommendation, moderate‐quality evidence (2B)

In adult patients with cardiac arrest in the OR, if ventricular fibrillation is present, immediate defibrillation should be carried out. If asystole is diagnosed, start closed chest compressions. If p-waves exist, temporary pacing may be tried if immediate available [42]. Closed chest compressions should be started immediately in patients who have cardiac arrest and intra-arterial blood pressure monitoring indicates no cardiac output. In cases of cardiac arrest due to hypovolaemia, tension pneumothorax or cardiac tamponade, CCC takes a lower priority than the immediate treatment of these reversible causes [43]. These causes usually develop gradually after a period of severe hypotension (peri-arrest state), which initially may present as pulseless electrical activity and if left untreated, becomes asystole.

#### Haemorrhage

- We suggest simultaneous haemorrhage control, massive transfusion, and closed chest compressions.
- Strong recommendation, low quality evidence (1C)
- We suggest simultaneous volume replacement and closed chest compression.
- Weak recommendation, low quality evidence (2C)
- If there is no return of spontaneous circulation despite adequate volume therapy, open chest cardiac massage may be considered.
- Weak recommendation, low‐quality evidence (2C)

There is currently little evidence [44] to support the routine use of OCCM in cardiac arrest due to massive haemorrhage. The routine use of OCCM in patients with massive haemorrhage requires re-evaluation.

Patients with hypovolaemic cardiac arrest are fundamentally different from those with primary cardiac arrest and, require different treatment. There are smaller increases in blood pressure when closed chest compression was used in the presence of reduced left ventricular volumes as seen in hypovolaemia. [45]. Results from some studies suggest that closed chest compressions confer no benefit when TCA is the result of haemorrhage [45, 46]. These results suggest that providing closed chest compressions when there is insufficient preload is likely to be futile.

In animal studies with different models of cardiac arrest, it has been found that CPR results in lower mean arterial and systemic perfusion pressures when compared with OCCM [47–49]. When cardiac arrest is due to massive haemorrhage OCCM may be considered as an option when CCC and fluid replacement do not result in ROSC. This technique requires training, experience and equipment.

### Resuscitative endovascular balloon occlusion of the aorta and resuscitative thoracotomy

In patients with exsanguinating infra-diaphragmatic haemorrhage uncontrollable by other means, we suggest either:

- Immediate use of resuscitative endovascular balloon occlusion of the aorta.
- Weak recommendation, low quality evidence (2C)

### OR

- Resuscitative thoracotomy with cross-clamping of the descending aorta or resuscitative endovascular balloon occlusion of the aorta.
- Weak recommendation, low quality evidence (2C)

Resuscitative thoracotomy and resuscitative endovascular balloon occlusion (REBOA) of the aorta are last interventions to occlude the descending aorta in patients with non-compressible, exsanguinating torso haemorrhage who are in cardiac arrest or peri-arrest. The key objectives of both procedures are to stop exsanguination and to maintain coronary and cerebral perfusion until definitive haemorrhage control is achieved. Due to the nature and the complexity of the underlying conditions solid evidence supported by randomised controlled trials is not available. Our recommendation is based on retrospective studies, evidence from prospective observational studies and recent authoritative guidelines on the topic. In summary we did not find any convincing superiority of either method over the other.

Many of the systematic reviews and meta-analyses that we found are based on the same pool of original publications. With few exceptions [50–52], most results indicate that REBOA yields a survival benefit over resuscitative thoracotomy or standard non-REBOA treatment. In a meta-analysis of REBOA versus resuscitative thoracotomy in blunt and penetrating trauma which included 1276 patients, a survival benefit was found for REBOA [53]. This finding was confirmed in another meta-analysis and systematic literature review. [54]

The aortic occlusion for resuscitation in trauma and acute care surgery registry of the American Association for the Surgery for Trauma compared outcomes in patients who have undergone REBOA versus cross-clamping of the aorta. The results suggest that REBOA is associated with a significant survival benefit in trauma patients not requiring CCC [55]. In patients who have received CCC at any point during the resuscitation such a benefit could not be confirmed.

The international aortic balloon occlusion register collects data on patients who have undergone REBOA for traumatic shock. They found a survival benefit for non-continuous over continuous REBOA. They also found complications related to ischaemia only in patients who had received continuous REBOA [56].

Aortic occlusion, whether by REBOA or resuscitative thoracotomy with cross clamping of the aorta, is a high-risk procedure and needs to be embedded into a well-established and rehearsed care pathway to ensure that ischaemic time is kept to a minimum and definitive haemorrhage control is carried out without delay. Aortic occlusion is not recommended if immediate access to definitive haemorrhage control is not available. [57]

As most studies are retrospective and confounded by significant selection, inclusion and survivor bias [57], these factors may explain the statistical superiority of REBOA. In cardiac arrest or peri-arrest situations associated with a poor outcome, clinicians would tend to proceed to resuscitative thoracotomy and aortic cross clamping instead of REBOA. Some investigators have tried to reduce this bias by introducing propensity score matching [58] but given the complexity of trauma resuscitation, it is not possible to control for all the confounding issues. We cannot recommend REBOA over resuscitative thoracotomy with aortic cross clamping.

### Tension pneumothorax

- We recommend immediate decompression of suspected tension pneumothorax.
- Strong recommendation, low quality evidence (1C)
- We recommend needle decompression immediately if tension pneumothorax is the proven or suspected cause of the cardiac arrest.
- Strong recommendation, low quality evidence (1C)
- We recommend finger thoracotomy or a chest tube insertion after any needle decompression attempt.
- Strong recommendation, low quality evidence (1C)

Increased intrathoracic pressure which obstructs venous return and results in mediastinal shift from the presence can cause a cardiac arrest. Tension pneumothorax (TPT) is a reversible cause of cardiac arrest that must be excluded during CPR. TPT may be caused by trauma, asthma and other respiratory disease, but can also be iatrogenic following invasive procedures, e.g. central line insertion, positive pressure ventilation, an unrecognised closed expiratory valve, or equipment failure. The institution of positive pressure ventilation can convert a simple pneumothorax into a TPT, particularly in patients with chest trauma [59] and severe asthma. The prevalence of TPT is approximately 0.5% [60] in all major trauma in the prehospital setting and in 13% of those developing traumatic cardiac arrest [61].

The diagnosis of TPT in a patient with cardiac arrest or haemodynamic instability must be based on clinical examination or point-of-care ultrasound [62]. The symptoms include hypotension or cardiac arrest in conjunction with signs suggestive of a pneumothorax (respiratory distress, hypoxia, absent unilateral breath sounds, subcutaneous emphysema and mediastinal shift (tracheal deviation and jugular venous distention) [59]. During CPR, not all of these signs may be present. When it is suspected in the presence of a cardiac arrest or severe hypotension, chest decompression should be carried out immediately [62] before radiographic confirmation [63]. Also, numerous studies note that lung ultrasound is one of the best means of accurately diagnosing a pneumothorax. While time is of the essence, point-of-care ultrasound should be considered [64].

In ventilated patients TPT presents rapidly with signs of respiratory and cardiac compromise. The incidence of cardiac arrest is significantly higher than in spontaneously breathing patients [59]. Rising ventilator pressures, reduced air entry and haemodynamic compromise should alert the clinician to the possibility of a TPT. Immediate thoracic decompression should be performed. The technique employed will depend on the available technical skills and access to the patient.

Decompression of the chest effectively treats TPT in patients with TCA and takes priority over all other measures. Finger thoracostomy is easy to perform and is used routinely in the prehospital field [67]. This step is the first stage of standard chest tube insertion – a simple incision and rapid dissection into the pleural space (see TCA, and Appendix 2). Chest tube insertion requires additional equipment, takes longer to perform and creates a closed system that has the potential for building retention inside of the thorax. Chest drain tubes may become blocked with lung or blood clots and have the potential to kink.

### Cardiac tamponade

- In suspected cardiac tamponade, point-of-care ultrasound should be used to confirm the diagnosis.
- Strong recommendation, low – quality evidence (1C)

This recommendation is based on a systematic review of retrospective evidence [68], the guidelines of the European Society for Cardiology, the Diagnosis and Management of Pericardial Disease [69] and one animal experimental study [45].

Point-of-care ultrasound is recommended to confirm the extent of cardiac tamponade and the resulting effect on haemodynamics. Causes of pericardial tamponade can be divided into surgical (mostly acute onset) and medical (mostly chronic). Cardiac tamponade in the perioperative period may develop after cardiac surgery, percutaneous cardiac interventions, central venous cannulations, laparoscopic surgery, radiofrequency ablation for hepatocellular carcinoma or in patients presenting for aortic dissection surgery [70–74].

Today cardiac tamponade is recognised as an essential diagnosis to exclude as a reversible cause during CPR. Ultrasound should be the principal diagnostic test to confirm pericardial tamponade and should be used to guide pericardiocentesis.

### Pericardiocentesis

- In case of cardiac tamponade, we recommend immediate decompression of the pericardium.
- Strong recommendation, low – quality evidence (1C)
- Immediate decompression can be achieved by either ultrasound guided pericardiocentesis or, in the case of a haemopericardium, by resuscitative thoracotomy.
- Strong recommendation, low – quality evidence (1C)

Needle pericardiocentesis under ultrasound guidance and resuscitative thoracotomy are the cornerstones of treatment for pericardial tamponade of non-traumatic origin [69]. In trauma, needle pericardiocentesis has been replaced by resuscitative thoracotomy and has virtually disappeared from clinical practice in the treatment of pericardial tamponade. This change of practice in TCA has come about because the pericardial blood collection is frequently clotted and cannot be aspirated by needle pericardiocentesis [68]. However, there may be a role for needle pericardiocentesis and catheter insertion as bridging measures before definitive surgical repair in severely compromised patients if resuscitative thoracotomy is not immediately available [100]. In order to avoid complications such as cardiac perforation, tension pneumothorax etc., needle pericardiocentesis should be carried out under ultrasound guidance [69, 101].

If pericardial tamponade has caused cardiac arrest, chest compressions are not effective. In hypovolaemic cardiac arrest, the circulatory collapse is caused by a lack of preload. Chest compressions further increase intrathoracic pressure and reduce venous return to the heart. Asynchronous chest compressions also hamper ventricular filling. Both factors compromise cardiac output [45], particularly during positive pressure ventilation [102]. Volume expansion with intravenous fluids [103] and immediate relief of the tamponade therefore take priority over chest compressions.

### Preparational aspects of cardiac arrest

#### Cardiac arrest team training

- When training for peri-operative cardiac arrest we suggest a co-ordinated protocol to improve the quality of mechanical cardiopulmonary resuscitation.
- Weak recommendation, low‐quality evidence (2C)

In a prospective, before-after cohort evaluation [104], the implementation of cardiac arrest team training incorporating an automated chest compression device (ACCD) resulted in a decrease of the no-flow ratio from 0.42 to 0.27 (95% CI, 0.10 to 0.19, *P* < 0.005) and from 0.24 to 0.18 (95% CI, 0.01 to 0.11, *P* = 0.02) for the next 5 min. The mean time taken to apply the ACCD decreased from 208.8 s to 141.6 s (decrease = 67.2 s, 95% CI, 22.3 to 112.1 s, *P* < 0.005).

The ACCD generated more consistent and higher systemic pressures and flows compared with manual chest compressions. Initial no-flow time encountered when using the ACCD is usually due to the time taken to employ the device, poor co-ordination and time when CPR is not performed. Being trained in a co-ordinated protocol improved the quality of mechanical cardiopulmonary resuscitation.

### Training of healthcare providers

- We suggest simulation training since experience and training of healthcare providers increases the likelihood of the return of spontaneous circulation.
- Weak recommendation, low‐quality evidence (2C)

A prospective, non-randomised study suggested that a trained CPR team increased the likelihood of return of spontaneous circulation (odds ratio = 8.76; 95% confidence interval, 2.5 to 30.72; *P* < 0.001) [105].

A decrease in mortality is seen when CPR is performed on patients suffering from cardiac arrest. Factors correlating with successful CPR are individual knowledge, skills, and training [106, 107]. The majority of studies were conducted in single hospital settings, making generalisation difficult. Moreover, changes in guidelines over time may affect future results.

### Clinical best practice statements

Hypovolaemic cardiac arrest.

- Point of care ultrasound has the potential to target resuscitative efforts in a cardiac arrest situation for assessment of volume and myocardial contractility

In hypovolaemic cardiac arrest, haemorrhage control and replacement of blood products take priority over chest compression. We have not found any evidence to support chest-compression in hypovolaemic cardiac arrest. In contrast, experimental animal studies suggest that chest compressions in hypovolaemic cardiac arrest further reduces cardiac output [45, 46, 49], and is associated with a significantly lower survival rate than resuscitation with fluid or blood products only [46, 49].

There is an essential difference between medical cardiac arrest and hypovolaemic cardiac arrest. The latter is caused by lack of cardiac preload and is preceded by hypovolaemic shock degrading into a minimal cardiac output state. The corresponding cardiac activity usually is pseudo-pulseless electrical activity (PEA) [108], in which there is insufficient co-ordinated cardiac activity to maintain signs of life. At this stage CCC should be withheld and resuscitation should focus on haemorrhage control and replacement of fluids and blood products, because the increase in intrathoracic pressure caused by CCC decreases venous return, and the asynchronous compression of the empty heart impedes diastolic ventricular filling, both further compromising cardiac output [49]. If left untreated cardiac contractions cease completely and true PEA ensues, which subsequently deteriorates into asystole.

Point of care ultrasound is strongly recommended [109] to:

- Detect the cause of hypovolaemic cardiac arrest
- Differentiate between pseudo- and true PEA
- Target resuscitative efforts
- Rule out other reversible causes

The transition from pseudo-PEA to PEA seems to be the 'point of no return' where survival rates drop below 1% [109, 110], despite aggressive resuscitation.

If cardiac activity does not resume after attempting correction of hypovolaemia, chest compressions or OCCM are indicated, particularly if the no/low flow times are short. If there is no immediate return of spontaneous circulation with CCC or OCCM and all other reversible causes are addressed, termination of resuscitation is justified.

### Withdrawing therapy

No specific evidence supporting recommendation for withdrawing therapy or immediate transfer to the intensive care unit, besides existing guidelines, was found.

Due to the lack of evidence, for adult patients suffering cardiac arrest in the operating theatre no recommendation can be made for withdrawing therapy or for their immediate transfer to intensive care unit. Current treatment strategies including extracorporeal cardiopulmonary resuscitation represent clinical practice without clear outcome prediction. [111]

Recent data have shown effective treatment of intraoperative cardiac arrest with good neurological outcome [112–114]. One retrospective observational study on refractory intraoperative cardiac arrest in non-cardiac surgery from one institution [111], and large database analysis of perioperative outcome in cardiac surgery [115] do not support any specific aspects of withdrawal therapy or resuscitation termination strategy for cardiac arrest in the operating room [116].

## Conclusions

This consensus guideline summarises recommendations of specific aspects of peri-operative cardiac arrest in respect of preparation, early identification, management, and treatment. Cardiac arrest in the operating room environment is characterised by the combination of reversible causes, the presence of highly trained staff, and well-equipped infrastructural resources. Under these conditions, this evidence-based guideline aims to complement the ALS guidelines of the ERC.

### Supplementary Information

Below is the link to the electronic supplementary material.

Supplementary file1 (DOCX 15 KB)

Supplementary file2 (DOCX 16 KB)

## Data Availability

Not applicable.
