# Supplementary material for: Cardiac arrest in the perioperative period: a consensus guideline for identification, treatment, and prevention from the European Society of Anaesthesiology and Intensive Care and the European Society for Trauma and Emergency Surgery
Source: Eur J Trauma Emerg Surg. 2023 Jul 10;49(5):2031–46. doi: 10.1007/s00068-023-02271-3 (PMC10520188; doi:10.1007/s00068-023-02271-3)
Supplement: Supplementary file 1 — Supplementary file1 (DOCX 15 KB) [file 68_2023_2271_MOESM1_ESM.docx]

**Appendix 1**

**GRADE recommendations**

| Grade of Recommendation | Clarity of risk/benefit | Quality of supporting evidence |
| --- | --- | --- |
| 1A.  Strong recommendation, high quality evidence | Benefits clearly outweigh risk and burdens, or vice versa. | Consistent evidence from well performed randomised, controlled trials or over-whelming evidence of some other form. Further research is unlikely to change our confidence in the estimate of benefit and risk. |
| 1B.  Strong recommendation, moderate quality evidence | Benefits clearly outweigh risk and burdens, or vice versa. | Evidence from randomised, controlled trials with important limitations (inconsistent results, methodologic flaws, indirect or imprecise), or very strong evidence of some other research design. Further research (if performed) is likely to have an impact on our confidence in the estimate of benefit and risk and may change the estimate. |
| 1C.  Strong recommendation, low quality evidence | Benefits appear to outweigh risk and burdens, or vice versa. | Evidence from observational studies, unsystematic clinical experience, or from randomised, controlled trials with serious flaws. Any estimate of effect is uncertain. |

| 2A.  Weak recommendation = suggestion, high quality evidence | Benefits closely balanced with risks and burdens. | Consistent evidence from well performed randomised, controlled trials or overwhelming evidence of some other form. Further research is unlikely to change our confidence in the estimate of benefit and risk. |
| --- | --- | --- |
| 2B.  Weak recommendation = suggestion, moderate quality evidence | Benefits closely balanced with risks and burdens, some uncertainly in the estimates of benefits, risks and burdens. | Evidence from randomised, controlled trials with important limitations  (inconsistent results, methodologic flaws, indirect or imprecise), or very strong evidence of some other research design. Further research (if performed) is likely to have an impact on our confidence in the estimate of benefit and risk and may change the estimate. |
| 2C.  Weak recommendation = suggestion, low quality evidence | Uncertainty in the estimates of benefits, risks, and burdens; benefits may be closely balanced with risks and burdens. | Evidence from observational studies, unsystematic clinical experience, or from randomised, controlled trials with serious flaws. Any estimate of effect is uncertain. |

<https://www.wolterskluwer.com/en/solutions/uptodate/policies-legal/grading-guide#:~:text=UpToDate%20has%20chosen%20a%20three,)%20(see%20table%201>
