# Supplementary material for: Cardiac arrest in the perioperative period: a consensus guideline for identification, treatment, and prevention from the European Society of Anaesthesiology and Intensive Care and the European Society for Trauma and Emergency Surgery
Source: Eur J Trauma Emerg Surg. 2023 Jul 10;49(5):2031–46. doi: 10.1007/s00068-023-02271-3 (PMC10520188; doi:10.1007/s00068-023-02271-3)
Supplement: Supplementary file 2 — Supplementary file2 (DOCX 16 KB) [file 68_2023_2271_MOESM2_ESM.docx]

**Appendix 2**

Tension pneumothorax

Immediate thoracic decompression should be performed in order of preference by finger thoracostomy in the 4^th^ or 5^th^ intercostal space 1cm anterior to the axillary line,^65^ by needle decompression in the 4^th^ intercostal space at the anterior axillary line,^66^ or needle decompression in the 2^nd^ intercostal space at the mid clavicular line.^66^ Needle decompression should be performed with an 8cm needle/cannula and a minimum diameter of 14G which should be inserted perpendicular to the chest wall above the superior border of the rib below. It should be inserted to the hub of the cannula and held in place for 5 to 10 seconds to allow decompression of the pleural space before removing the needle from the cannula.

A finger thoracostomy is the treatment of choice for traumatic cardiac arrest resulting from a tension pneumothorax,^6^ this requires the patient to be positioned supine, the arm to be abducted, an appropriately trained person to perform the procedure as well as the availability of basic equipment (e.g. scalpel and forceps). It will ensure that the pleural cavity has been entered and decompressed and permits insertion of a tube thoracostomy drain to allow the lung to re-expand. If neither the equipment nor an appropriately trained individual is immediately available, needle decompression should be performed via the axilla (4^th^ or 5^th^ intercostal space 1cm anterior to the mid axillary line). This is preferable to using the mid clavicular line. Numerous studies have shown that the chest wall is significantly thinner in the axilla and failure rates using this approach are significantly lower.^3-7^ This technique can also be used in a patient positioned prone.

If the axilla is not immediately accessible needle decompression should be performed in the 2^nd^ intercostal space in the mid clavicular line rather than delay decompression to reposition the patient. An 8 cm needle/cannula set should be long enough to reach the pleural cavity in most patients.^66^ While some authors recommend using a 10G needle,^67^ most report successful treatment with a 14G. It should be inserted perpendicular to the chest wall to minimise the amount of tissue it has to pass through, and above the superior border of the rib below to minimise the risk of injury to the neurovascular bundle. By holding the needle/cannula in place until the lung has been decompressed (5-10 seconds) before removing the needle the effect of the cannula kinking will be reduced. Leaving the needle in situ after the lung has expanded increases the risk of iatrogenic injury.
